# Caries Spine Presenting as Thyroid and Multiple Paraspinal Abscesses

**DOI:** 10.4084/MJHID.2010.008

**Published:** 2010-05-04

**Authors:** Adnan Agha, Mohammad Awad AlHumaidi, Abdelhaleem Bella

**Affiliations:** Department of Internal Medicine, King Fahad Hospital, Armed Forces Hospital Program Southern Region, Khamis Mushyt, P.O. Box 101, Kingdom of Saudi Arabia

**Figure:**
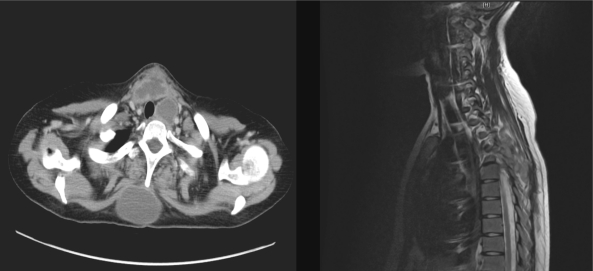


## Description:

This 21 years old women presented consecutive cough with sputum and intermittent high grade fever all through her last trimester of pregnancy. On examination she was sick looking, febrile, and having bilateral lower limb weakness with spasticity and positive ankle clonus. She also had multiple swelling over upper back and neck without redness or hotness over it. The patient was admitted. The subsequent CT scan of the neck is significant for multiple cold abscesses all over body with one visible in the thyroid gland and one compressing on the thoracic spine. The cold abscesses were drained and spine decompression surgery was done. The patient was started on 4-drug regimen of anti-tuberculosis therapy and is now without complaints or any residual abscesses after four months.

